# Supplementary material for: Establishing predictive machine learning models for drug responses in patient derived cell culture
Source: NPJ Precis Oncol. 2025 Jun 13;9:180. doi: 10.1038/s41698-025-00937-2 (PMC12166088; doi:10.1038/s41698-025-00937-2)
Supplement: Supplementary file 1 — Supplementary materials [file 41698_2025_937_MOESM1_ESM.pdf]

Supplementary Information

Personalised Medicine – Establishing predictive machine learning models for drug responses in patient derived cell culture.

'cladribine', 'etoposide', 'gefitinib', 'bosutinib', 'ponatinib', 'clofarabine', 'cisplatin', 'oxaliplatin', 'paclitaxel', 'pazopanib', 'neratinib', 'melphalan', 'dasatinib', 'vinblastine', 'afatinib', 'teniposide', 'axitinib', 'lapatinib', 'nintedanib', 'vinorelbine', 'gemcitabine', 'sorafenib', 'erlotinib', 'cediranib', 'brigatinib', 'docetaxel', 'regorafenib', 'crizotinib', 'sunitinib', 'trametinib', 'topotecan', 'doxorubicin', 'vorinostat', 'vemurafenib', 'everolimus'

Table S1. Drugs included in the RX dataset.

Matrix completion

Given the incomplete nature of the datasets, even after the pruning described in the methods section, addressing missing values was a crucial aspect of this study. We considered two distinct approaches for handling these gaps. The first approach involved filling out the minority of missing values with zeros, resulting in what we refer to as the "Zeros-matrix". The alternative strategy was to utilise transformational machine learning to impute the values through predictive modelling, leading to the "TML-matrix".

In the case of the TML-matrix, we performed an experiment where 20 drugs were randomly selected for the panel, and a random forest regressor with 50 trees was employed as the learner. This experiment was conducted five times, and the outcomes are detailed in Table S2.

	$R_{pearson}$	$R_{spearman}$	*RMSE*
TML-matrix	0.770/0.020	0.783/0.020	0.500/0.027
Zeros-matrix	0.723/0.008	0.740/0.071	0.546/0.078

Table S2: Comparison of two approaches for addressing missing values within the GDSC1 dataset. Evaluation includes Pearson and Spearman correlation coefficients, along with RMSE. Results are presented as mean/stdev for clarity.

It became evident that the TML-matrix outperformed the Zeros-matrix. However, when accounting for standard deviations, substantial improvement was primarily observed in Pearson correlations. We therefore analysed the results further by assessing changes in Spearman R, focussing on any change exceeding 0.05 in any cell line (Figure S1).

While the TML matrix didn't significantly enhance performance for the majority of the 81 cell lines in the validation set, it showed the potential for substantial improvement in a smaller subset of cell lines without any notable downsides. Specifically, we found an improvement above 0.05 for 18 cell lines using the TML-matrix. The highest change in performance being 0.573 (a change of $R_{spearman}$ from 0.205 to 0.778). Of the 16 cell lines that performed better with the zeros-matrix, the improvements were marginal, ranging from 0.001 to 0.007. The average standard deviation for the Zeros-matrix across all cell lines was 0.067, in contrast to the 0.021 for the TML matrix. Given these findings, we made the decision to proceed with the TML matrix in our work.

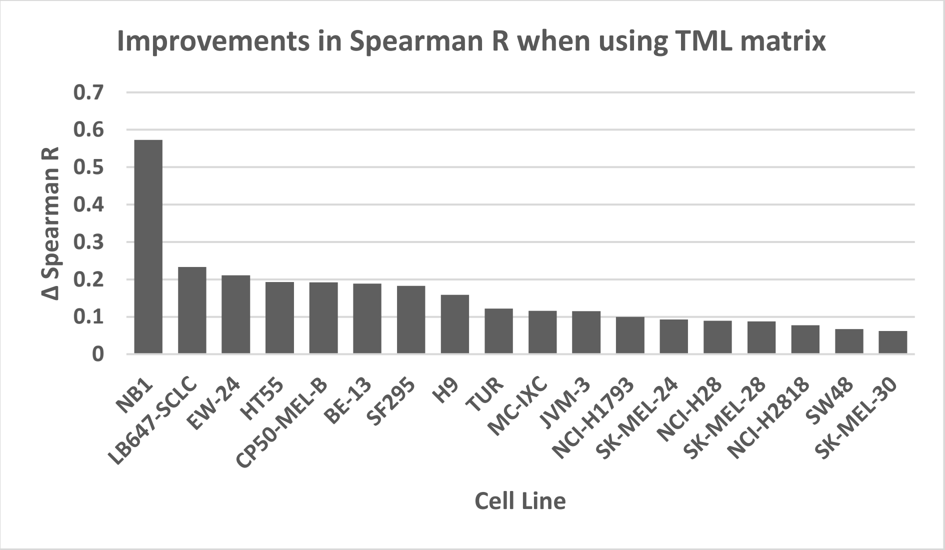

Figure S1. Depicts all changes to Spearman R values exceeding 0.05 when using the TML matrix. No negative changes above this threshold were observed.

Learner selection

The next objective was to determine which learner to use. We conducted experiments using both Random Forests and Gradient Boosting Trees, both of which has been extensively used for bioactivity prediction in literature. We utilised a panel of 20 randomly selected drugs and a training set of 100 randomly selected patients. We first explored variations in the number of trees. Notably we found no substantial improvement transitioning from 50 to 100 trees for either learner (Table S3). We also investigated increasing the depth for Gradient Boosting Trees from 1 to 3, and interestingly, found that a shallower depth resulted in better performance with this dataset. However, it became evident that Random Forests were better suited for this task. Consequently, we decided to proceed with Random Forests, employing a configuration of 50 trees.

Learner	$R_{pearson}$	$R_{spearman}$	*RMSE*
XGB-50-1D	0.738/0.028	0.751/0.028	0.518/0.027
XGB-100-1D	0.741/0.029	0.751/0.03	0.515/0.029
XGB-50-3D	0.705/0.042	0.719/0.044	0.545/0.038
XGB-100-3D	0.707/0.04	0.721/0.042	0.545/0.037
RF-50	0.757/0.022	0.770/0.021	0.510/0.028
RF-100	0.761/0.021	0.774/0.02	0.506/0.028

Table S3: Comparison of learner performance on GDSC1 dataset using a random selection of 20 drugs in the panel and 100 patients in the training set. Learners are indicated as follows: Learner (XGB/RF)-n-trees (50/100) – Depth for XGB (1/3). Default depth was used for RF. Evaluation includes Pearson and Spearman correlation coefficients, in addition to RMSE. Results are presented as mean/stdev for clarity.

Drug selection approach for probing panels

In our methodology, the selection of a probing drug panel to profile new cell lines is a critical aspect. We explored two different approaches to construct this panel. All patients in the training set were included in this experiment.

One approach involves randomly selecting several drugs from the library being probed. Resulting in a panel that generally mirrors the library's distribution. Alternatively, a panel can be created based on specific criteria, such as maximising the variability of drug responses. This can be achieved considering factors like substructures or functional properties.

In our selection procedure we began by excluding half of the library, specifically the portion with the lowest variability in responses. We iterated through the remaining drugs in random order, ultimately choosing 20 or 30 drugs with the lowest correlation in their responses when compared to all other drugs in this set. This approach allowed us to select drugs based not only on their variability in responses across cell lines, but also on their functional differences.

Our findings indicated that the randomly selected compounds performed slightly better on average, but the differences were marginal, especially when 30 drugs were selected (Table S4). However, it became clear that the standard deviations were larger for the randomly selected drugs. This was due to the variation between the runs and highlighted that random selection can yield both better and worse results. In contrast, the performance using the selected panel was more robust and remained competitive.

Another noteworthy observation is that while increasing the size of the drug panel to 30 drugs yielded better performance, a majority of the valuable information contained within this panel was effectively captured in the smaller 20-drug panel.

Panel (size)	$R_{pearson}$	$R_{spearman}$	*RMSE*
Selected (20)	0.767/0.008	0.783/0.008	0.497/0.009
Random (20)	0.789/0.020	0.799/0.020	0.458/0.024
Selected (30)	0.793/0.008	0.802/0.008	0.455/0.008
Random (30)	0.797/0.019	0.805/0.019	0.450/0.024

Table S4: Comparative evaluation of drug-panel selection procedures (selected or random), focussing on their impact on predictive performance. Size refers to the number of drugs included in the panel. Evaluation includes Pearson and Spearman correlation coefficients, in addition to RMSE. Results are reported as mean/stdev for clarity.

Drug panel size and its effect on model performance (GDSC1)

After establishing the selection procedure, we aimed to further explore how panel size affects predictive performance. To do this, repeated the selection procedure described above using the 40 most functionally diverse drugs from the more variable half of the drug library. To facilitate comparison across different panel sizes, we aimed to retain the same drugs. Therefore, we derived a panel of 30 drugs by selecting the 30 drugs with the least functional correlations amongst the 40 initial drugs. This process extended to create panels of sizes 20 and 10 as well, ensuring that each smaller panel represented a subset of the larger panel. This approach allowed us to systematically assess the impact of panel size on predictive performance.

Panel size	$R_{pearson}$	$R_{spearman}$	*RMSE*
10 drugs	0.737/0.018	0.753/0.018	0.548/0.020
20 drugs	0.775/0.010	0.783/0.010	0.481/0.012
30 drugs	0.796/0.008	0.805/0.008	0.446/0.008
40 drugs	0.806/0.007	0.812/0.007	0.436/0.008

Table S5: Investigating the effect of drug-panel size on predictive performance. Results include RMSE, as well as Pearson and Spearman correlation coefficients. Results are reported as mean/stdev for clarity.

Furthermore, our research delved into studying the correlation between the number of "historical samples" i.e., cell lines present in the training data, and the model's ability to predict drug responses in new samples. This correlation is of outmost importance for the practical feasibility of implementing this kind of protocol in a clinical setting. All the investigations were carried out using a dedicated validation set from the GDSC1 dataset, encompassing 81 patients, which accounts for 10% of all patients in the dataset.

Patient panel size in training set and its effect on model performance (GDSC1)

This methodology is dependent on two essential components, the number of drugs and the number of patient-derived cells available. While the drug space in precision medicine is limited to the number of approved drugs, the ideal number of patients necessary to establish an efficient model for predicting drug responses in new patients remains uncertain.

The GDSC1 dataset contains over 800 patients. 647 of which are not part of a validation or test set. We conducted a comparative analysis, evaluating performance when using all available patients and when working with smaller subsets, including 10, 30, 50, 100 and 200 patients. While utilising all available patients yielded the best results, the decrease in performance when working with smaller datasets were minimal. Notably, even when reducing this to 100 randomly selected patients, most of the valuable information remained intact (Table S6). In fact, reducing the set to 30 or even 10 patients still provided robust predictions not too far behind those achieved using the full dataset. After consideration, we decided to continue with 100 patients, as we deem that the increase in the dataset size did not justify the minor improvement in performance ($\Delta R_{pearson} = 0.02$).

patients (n)	$R_{pearson}$	$R_{spearman}$	*RMSE*
10	0.693/0.043	0.704/0.045	0.535/0.032
30	0.743/0.030	0.756/0.029	0.498/0.025
50	0.755/0.024	0.766/0.024	0.488/0.021
100	0.771/0.019	0.782/0.018	0.474/0.016
200	0.781/0.015	0.791/0.015	0.467/0.014
647 (all)	0.791/0.006	0.801/0.006	0.458/0.006

Table S6: Comparing performance with the all the patients available in the training set versus random subsets of the GDSC1 dataset. RMSE, as well as Pearson and Spearman correlation coefficients are reported. Results are presented as mean/stdev.

GDSC1 performance histogram

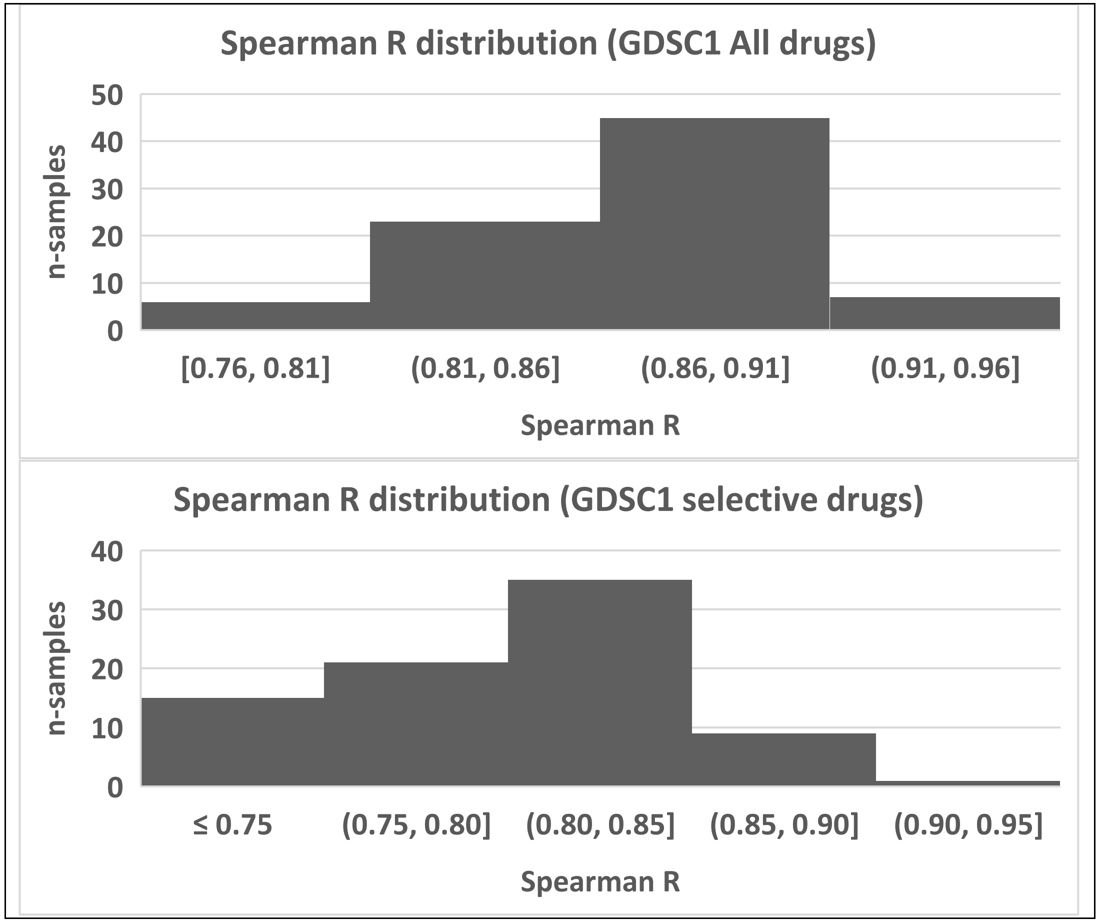

Figure S2. Histogram showing the distribution of Spearman R across 81 cell lines for all drugs as well as selective drugs.

Patient panel size used in training set and impact on model performance (PRISM)

Patients(n)	$R_{pearson}$	$R_{spearman}$	*RMSE*	*top 0.5%*	*top 1%*	*top 2%*	*Hit rate*
10	0.596/0.040	0.378/0.031	0.714/0.019	0.097/0.057	0.218/0.088	0.452/0.110	0.695/0.130
30	0.653/0.028	0.437/0.023	0.693/0.014	0.125/0.063	0.287/0.075	0.522/0.071	0.782/0.081
50	0.668/0.021	0.452/0.020	0.686/0.010	0.150/0.063	0.329/0.063	0.551/0.052	0.814/0.058
100	0.686/0.016	0.473/0.015	0.685/0.009	0.173/0.061	0.358/0.057	0.577/0.040	0.858/0.050
200	0.696/0.012	0.486/0.012	0.680/0.007	0.178/0.057	0.370/0.053	0.586/0.035	0.870/0.0370
all (418)	0.690/0.011	0.473/0.008	0.682/0.008	0.163/0.060	0.343/0.059	0.569/0.041	0.844/0.047

Table S7: Effect of patient sample size in training set on prediction performance. The reported metrics include $R_{pearson}$, $R_{spearman}$, RMSE, accuracy of predictions within the top 10, 20 and 30 drugs, and hit-rates among the top 10 recommendations. Results are presented as mean/stdev derived from five experiments using the PRISM dataset (validation subset used for these experiments).

Drug panel size and its impact on model performance (PRISM)

panel size	$R_{pearson}$	$R_{spearman}$	*RMSE*	*top 0.5%*	*top 1%*	*top 2%*	*Hit rate (top 45)*
23 drugs	0.522/0.036	0.391/0.025	0.779/0.010	0.048/0.029	0.135/0.051	0.332/0.085	0.534/0.094
45 drugs	0.611/0.019	0.446/0.013	0.742/0.009	0.080/0.046	0.197/0.058	0.420/0.067	0.662/0.077
90 drugs	0.690/0.011	0.473/0.008	0.682/0.008	0.163/0.060	0.343/0.059	0.569/0.041	0.844/0.047

Table S8: Effect of panel size on predictive performance using either 23, 45 or 90 drugs. A training set of 100 randomly selected patients were used. The reported metrics encompass $R_{pearson}$, $R_{spearman}$, RMSE, accuracy of predictions within the top 10, 20 and 30 drugs, and hit-rates among the top 10 recommendations. Results are presented as mean/stdev derived from five experiments. PRISM dataset was used (validation subset employed to derive results).

Relationship between the cancer tissue types in training data and predictive performance

Existing research suggests that stratifying patient-derived cells can improve drug response prediction[30]. Recognising that patient clustering is dynamic and varies based on the drugs of interest, we investigated this further. First, we compared the performance of random selection of 10 patients from the entire cohort versus restricting the cohort to the same tissue type, including half the drug library in the drug panel. The results showed similar performance, suggesting that drug response prediction is not necessarily dependent on having a large cohort of patients with the same cancer type (Figure S3). This is further illustrated in Figure 4 of the main text, showing the composition of the ten most informative cancer tissue types when predicting example cell lines from different tissues of origin.

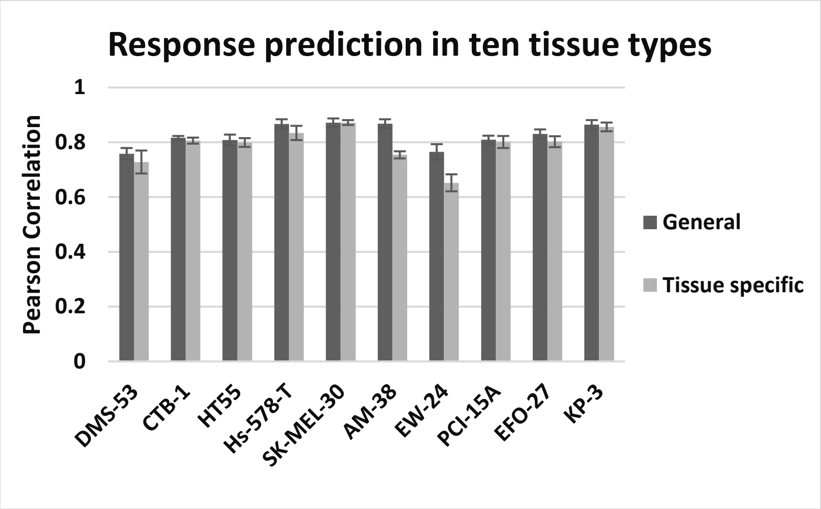

Figure S3. Comparative analysis between using ten randomly selected cells from all (General) vs. only tissue specific cell-lines in training sets from the GDSC1 dataset. Pearson correlations reported for ten different cell lines with diverse tissue origins.

Effect of Drug Panel size and composition on patient response clustering

We further study how patients cluster together depending on the drugs used in the panel (Figure S4). To this end, we first selected four cell lines with different tissue origins to investigate (lung, breast, skin and pancreas). We then randomly selected a drug panel for four different size thresholds (10, 30, 50 and 80 percent of the GDSC1 library). We generated random forest models as described above and extracted the 10 most important features (other cell lines that are influential for accurate prediction). Amongst the 10 most featured cell lines across 10 experiments, we counted the number of times the training data cell lines made appearance as a top 10 feature. For instance, in the case of the lung cancer cell line, you'll find that the top featured cell line (number 1), appeared as a top10 feature in 4 out of 10 experiments when 10% (26 drugs) of the drug library was used as a probing panel. In contrast, it featured as a top10 feature in every experiment when 50% (133 drugs) of the drugs were included in the probing panel.

In essence, we found that the selection of drugs that is included in the panel (as it is randomly selected in each experiment) and the size of the panel, have a profound impact on how patients cluster. The clustering becomes more robust when increasing the size of the panels, suggesting that overall, specific cell lines tend to be similar in their general response (hints at the concept of digital twins in precision medicine). However, within confined drug spaces, as represented by the random selection of drugs in the smaller panels, a cell line can overlap with a variety of cell lines in their responses. We are not focussed on performance in this analysis, and we are aware that larger panels should result in better performance (Table S5). However, we have already confirmed that even smaller panel sizes than the minimum 10 percent (26 compounds) used here results in strong performance, and that random selection does not cause significant alterations to the performance compared to a selection protocol (Table S4). Hence these results are robust in the context of our analysis.

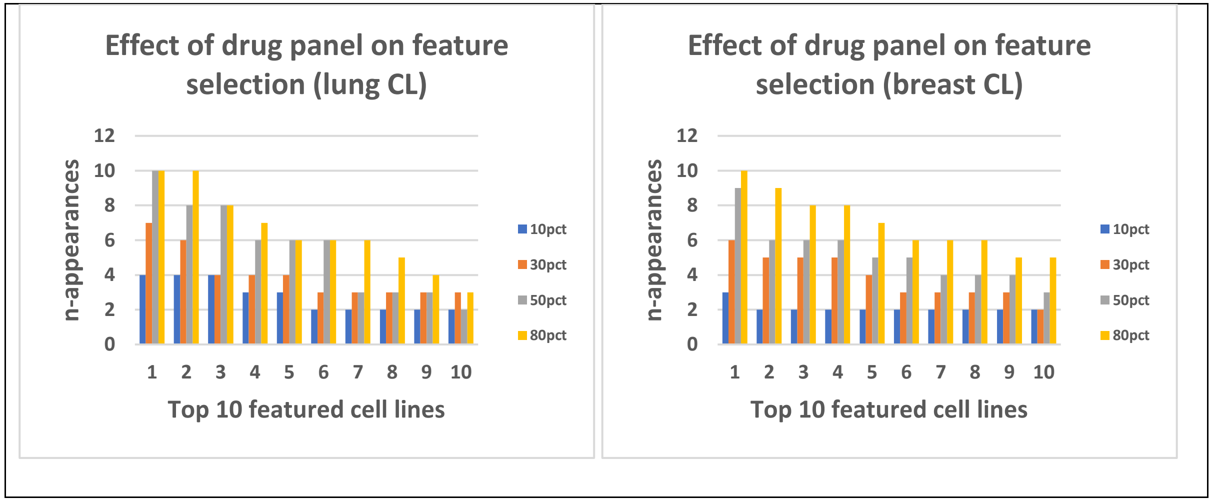

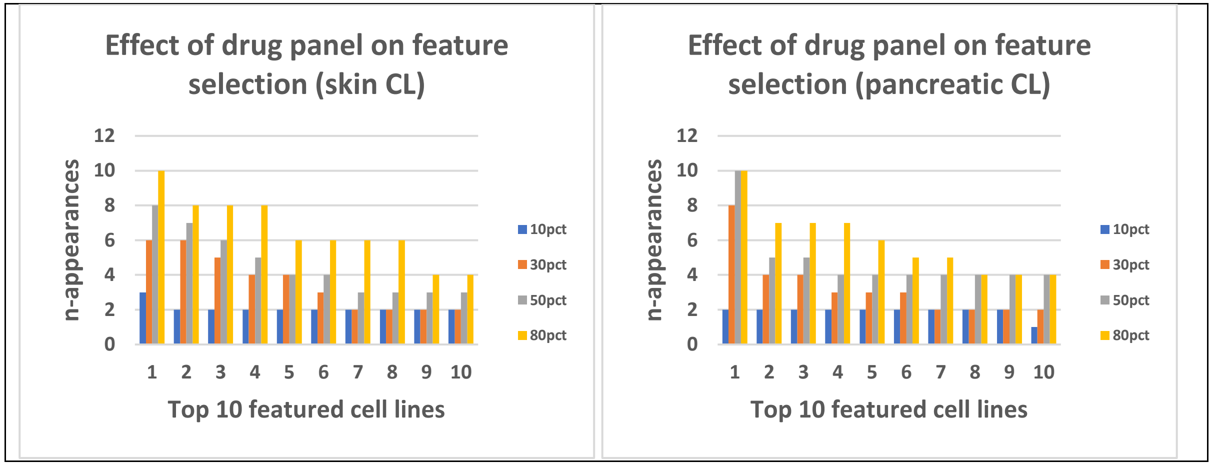

Figure S4. Impact of drug panel size (and composition) on the most important features selected to predict four different cell lines of different tissue origins. The results are presented as the number of times the 10 most featured cell lines are present in the top10 most influential features out of 10 experiments. This was done for four different drug panel size thresholds: 10, 30, 50 and 80% of the entire GDSC1 drug library.

Scenario exploring how effectively the methodology would be able to identify hits and at least one top3 drug when testing the top 3 predictions.

HITS	ALL (%)	SELECTIVE (%)
AT LEAST 1	100/0.00	79.8/0.03
AT LEAST 2	100/0.00	51.4/0.07
AT LEAST 3	97.1/0.02	23.4/0.08
A TOP 3 DRUG IDENTIFIED	73.7/0.01	43.2/0.05

Table S9. Assessment of the methodology's performance in identifying hits and capturing at least one actual top-3 drug within the top-3 predictions. Results are shown for two scenarios: (1) all drugs and (2) selective drugs. For each scenario, we report the number of hits (1, 2, or 3) and the percentage of cases where at least one actual top-3 drug was correctly predicted. 200 samples were randomly selected for training. Values represent the mean percentage across three experiments ± standard deviation.

Scenario exploring how effectively the methodology would be able to catch the top 1 and 3 most potent drugs in the library against each cell line in the test set when deciding to test the top 15.

COMPOUNDS	ALL (%)	SELECTIVE (%)
**1 OUT OF TOP 3 DRUGS**	100/0.00	82.7/0.03
**2 OUT OF TOP 3 DRUGS**	92.2/0.01	49.4/0.02
**3 OUT OF TOP 3 DRUGS**	67.1/0.02	17.3/0.01
**OPTIMAL DRUG IDENTIFIED**	79.0/0.03	56.8/0.03

Table S10. Evaluation of the methodology's ability to capture top-3 drugs within the top-15 predictions. For each scenario (1) all drugs and (2) selective drugs, we report the number of actual top-3 drugs identified (1, 2, or 3) and whether the best-performing drug was correctly predicted. 200 samples were randomly selected for training. Results are presented as the mean percentage of cases across three experiments ± standard deviation.

Parallel comparison of our approach with MACCS fingerprints

PANEL SIZE	HTSFP $R_{PEARSON}$	MACCS $R_{PEARSON}$	HTSFP $R_{SPEARMAN}$	MACCS $R_{SPEARMAN}$	HTSFP RMSE	MACCS RMSE
**30 DRUGS**	0.725/0.033	0.081/0.023	0.740/0.034	0.078/0.025	0.537/0.029	0.812/0.010
**100 DRUGS**	0.755/0.031	0.147/0.032	0.756/0.033	0.132/0.033	0.501/0.027	0.779/0.010
**150 DRUGS**	0.772/0.040	0.167/0.044	0.764/0.039	0.141/0.046	0.481/0.038	0.772/0.013
**190 DRUGS**	0.755/0.075	0.212/0.096	0.714/0.090	0.188/0.111	0.482/0.067	0.758/0.027

Table S11. Comparing our approach (HTSFP) with molecular fingerprints (MACCS) in predictive performance on the GDSC1 test set. 10 were patients used to construct the HTSFP. Varying panel size with 30-190 drugs used by our approach are shown. The remainder of 202 drugs was selected as the test set in each experiment. Results reported as mean/stdev for each metric.

patient	All drugs mean viability	Top 5 ranked mean viability	Total hits available	Drugs tested	Hits identified	Total n drugs available	Spearman R all drugs
P1	76.17	27.92/0.00	3	5.0/0.0	3.0/0.0	34	0.57/0.02
P2	86.06	5.62/0.79	6	6.4/0.5	6.0/0.0	35	0.73/0.04
P3	115.16	65.40/7.79	2	3.0/0.0	0.2/0.4	34	0.57/0.02
P4	92.19	41.10/0.00	3	2.0/0.0	2.0/0.0	34	0.59/0.01
P5	96.1	35.79/0.40	3	4.0/0.0	3.0/0.0	34	0.48/0.02
P6	101.12	53.92/0.00	0	N/A	N/A	32	0.61/0.01
P7	97.43	80.00/0.68	1	0.0/0.0	0.0/0.0	33	0.47/0.01
P8	108.21	50.80/4.84	2	1.4/0.5	1.4/0.5	34	0.67/0.01
P9	71.06	3.55/3.76	6	7.6/0.5	6.0/0.0	34	0.77/0.01
P10	96.37	66.05/0.00	2	1.0/0.6	1.0/0.6	34	0.29/0.02
P11	78.78	22.39/0.00	3	3.4/0.5	2.0/0.0	33	0.80/0.01
P12	115.41	35.15/1.21	3	3.0/0.0	3.0/0.0	30	0.61/0.01
P13	95.57	49.99/0.00	2	1.2/0.4	0.2/0.4	35	0.75/0.02
P14	101.48	34.35/6.38	3	2.6/0.5	2.0/0.0	33	0.35/0.02
P15	76.66	18.10/4.43	3	7.8/0.8	3.0/0.0	33	0.68/0.01
P16	90.96	58.21/4.59	0	N/A	N/A	33	0.54/0.01
P17	107.69	89.84/3.88	0	N/A	N/A	32	0.45/0.01
P18	55.43	-3.13/3.02	11	12.0/0.0	10.0/0.0	33	0.77/0.01
P19	87.94	4.70/0.00	7	5.2/0.4	5.0/0.0	31	0.54/0.08
P20	81.96	10.56/4.32	6	8.6/0.5	6.0/0.0	32	0.74/0.01
P21	76.49	25.96/4.05	4	5.8/0.4	3.4/0.5	31	0.70/0.01
P22	97.2	69.33/7.70	1	0.0/0.0	0.0/0.0	31	0.31/0.03
P23	42.88	-20.64/3.70	15	15.0/0.0	15.0/0.0	33	0.93/0.01
P24	52.8	-22.08/6.38	10	11.2/0.4	7.0/0.0	33	0.87/0.01
Mean	87.55	33.45/2.83	4.36	4.87/0.27	3.60/0.11	33	0.62/0.02

Table S12. Results (average/standard deviation) for each patient in the RX dataset. Each experiment was performed five times using GradientBoostingRegressor (n_estimators = 50, max_depth=5). The average viability resulting from the drug library is reported, along with that found when selecting the top 5 predicted drugs. Defined as reducing cell viability to less than thirty percent, the average number of hits available per cell line is shown, as well as the average number of drugs predicted to be hits, and the average number of these correctly identified as such. The average number of drugs tested per cell line is found in the last column “drugs in panel”.
